# Inhibitors of One or More Cellular Aurora Kinases Impair the Replication of Herpes Simplex Virus 1 and Other DNA and RNA Viruses with Diverse Genomes and Life Cycles

**DOI:** 10.1128/spectrum.01943-22

**Published:** 2022-12-20

**Authors:** Cindy Y. Ly, Jessica Pfannenstiel, Anil Pant, Zhilong Yang, Anthony R. Fehr, Maxim S. Rodzkin, David J. Davido

**Affiliations:** a Department of Molecular Biosciences, University of Kansas, Lawrence, Kansas, USA; b Division of Biology, Kansas State University, Manhattan, Kansas, USA; c Department of Veterinary Pathobiology, College of Veterinary Medicine & Biomedical Sciences, Texas A&M, College Station, Texas, USA; Barnard College, Columbia University

**Keywords:** herpesvirus, HSV-1, aurora kinases, kinase inhibitors, coronavirus, poxvirus

## Abstract

We utilized a high-throughput cell-based assay to screen several chemical libraries for inhibitors of herpes simplex virus 1 (HSV-1) gene expression. From this screen, four aurora kinase inhibitors were identified that potently reduced gene expression during HSV-1 lytic infection. HSV-1 is known to interact with cellular kinases to regulate gene expression by modulating the phosphorylation and/or activities of viral and cellular proteins. To date, the role of aurora kinases in HSV-1 lytic infection has not been reported. We demonstrated that three aurora kinase inhibitors strongly reduced the transcript levels of immediate-early (IE) genes ICP0, ICP4, and ICP27 and impaired HSV-1 protein expression from all classes of HSV-1, including ICP0, ICP4, ICP8, and gC. These restrictions caused by the aurora kinase inhibitors led to potent reductions in HSV-1 viral replication. The compounds TAK 901, JNJ 7706621, and PF 03814735 decreased HSV-1 titers by 4,500-, 13,200-, and 8,400-fold, respectively, when present in a low micromolar range. The antiviral activity of these compounds correlated with an apparent decrease in histone H3 phosphorylation at serine 10 (H3S10ph) during viral infection, suggesting that the phosphorylation status of H3 influences HSV-1 gene expression. Furthermore, we demonstrated that the aurora kinase inhibitors also impaired the replication of other RNA and DNA viruses. These inhibitors significantly reduced yields of vaccinia virus (a poxvirus, double-stranded DNA, cytoplasmic replication) and mouse hepatitis virus (a coronavirus, positive-sense single-strand RNA [ssRNA]), whereas vesicular stomatitis virus (rhabdovirus, negative-sense ssRNA) yields were unaffected. These results indicated that the activities of aurora kinases play pivotal roles in the life cycles of diverse viruses.

**IMPORTANCE** We have demonstrated that aurora kinases play a role during HSV-1 lytic infection. Three aurora kinase inhibitors significantly impaired HSV-1 immediate-early gene expression. This led to a potent reduction in HSV-1 protein expression and viral replication. Together, our results illustrate a novel role for aurora kinases in the HSV-1 lytic cycle and demonstrate that aurora kinase inhibitors can restrict HSV-1 replication. Furthermore, these aurora kinase inhibitors also reduced the replication of murine coronavirus and vaccinia virus, suggesting that multiple viral families utilize the aurora kinases for their own replication.

## INTRODUCTION

Protein kinases are critical in regulating many biological processes within a cell (1–4). Kinases are regulatory enzymes that facilitate a phosphate transfer, typically from ATP to target proteins. The phosphorylation triggers a conformational change in the target protein or creates a protein-protein interaction, affecting the target protein’s functions (2). Both DNA and RNA viruses exploit cellular kinases to modulate several aspects of their replication cycles, such as disruption of cell cycle, immune evasion, and regulation of transcription and replication (5–8). Influenza A virus (IAV) hijacks multiple cellular kinases to evade immune responses and aid in its replication (9–11). IAV upregulates c-Jun N-terminal kinase (JNK) to mediate the production of chemokines and cytokines (12). Human immunodeficiency virus (HIV) Tat protein binds directly to cdk9/cyclin T1 to recruit the transcription elongation complex to its genome during replication (13, 14).

The regulation of cellular kinases by herpes simplex virus 1 (HSV-1) is essential during its life cycle (15–20). Host cyclin-dependent kinases (CDKs) are required for transcription of HSV-1 genes and viral replication (21, 22). Previous studies have shown activation of CDK-1 is critical for the expression of HSV-1 late proteins during the HSV-1 lytic cycle (23). During HSV-1 reactivation, JNK facilitates a methyl-phospho switch on HSV lytic promoters, allowing viral gene expression to be stimulated (24). HSV-1 also encodes two viral kinases, US3 and UL13, and their kinase activities are critical in phosphorylating a variety of viral and cellular proteins during viral replication (25, 26). Taken together, these studies indicate that viral and host kinase activities are critical in modulating HSV-1 infection. While many reports have focused on specific cellular kinases, no study has examined an extensive or full repertoire of the 518 cellular kinases in the HSV-1 life cycle (27).

HSV-1 is a large double-stranded DNA virus with features of a glycoprotein-decorated envelope, an amorphous tegument protein layer, and a proteinaceous nucleocapsid. It infects about 80% of the global population as a lifelong obligate intracellular pathogen. HSV-1 can cause recurrent orofacial sores, herpes stromal keratitis, encephalitis, and life-threatening neonatal infections (28). Currently, there is no approved vaccine for HSV-1, and therapeutics remain limited to nucleoside analogs, such as acyclovir. Acyclovir-resistant strains of HSV-1 have been identified in the population, demonstrating the need to identify novel inhibitors of HSV-1 (29, 30).

Using a high-throughput screen, we have identified four potent aurora kinase inhibitors that restrict HSV-1 gene expression (31). The aurora kinase family collectively works together during mitosis to regulate the cell cycle and ensure proper chromosome segregation. Aurora A kinase is located at the spindle poles, regulating mitotic entry through centrosome maturation and stabilizing spindle assembly (32, 33). Aurora B kinase localizes at the kinetochores, ensuring proper chromosome segregation. Previous studies have shown aurora B kinase is the activating component to the chromosomal passenger complex (CPC) with inner centromere protein, Survivin, and Borelian proteins (33, 34). Together the aurora B/CPC complex is responsible for faithful execution of cytokinesis and phosphorylation of histone H3. While aurora A and B are found in cells throughout the body, aurora C kinase is limited to cells that undergo meiosis, and it remains understudied (33).

Aurora kinases have been explored in diverse viruses and found to have a broad range of functions. In hepatitis B virus, aurora A enhances viral transcription independent of its kinase activity during mitosis. The depletion of aurora A inhibited viral replication and gene expression (35). During dengue virus replication, aurora B kinase is involved in the viral morphogenesis or release of viral particles. Silencing of aurora B kinase leads to a significant decrease in viral yields (36). Epstein-Barr virus infection upregulates aurora B kinase expression by enhancing its promoter activity early during infection (37). The role of aurora kinases in HSV-1 productive infection has not been explored.

In this study, we investigated the contribution of aurora kinases during HSV-1 replication using three well-characterized aurora kinase inhibitors identified from a high-throughput screen. We observed a significant reduction in HSV-1 replication in a dose-dependent manner. Treatment with TAK 901, JNJ 7706621, or PF 03814735 decreased HSV-1 immediate-early (IE) transcript levels, which resulted in diminished IE protein expression. These results demonstrate that aurora kinases play a critical role in HSV-1 lytic infection related to viral transcription. The antiviral activities of these compounds correlate with a noticeable decrease in histone H3 phosphorylation at serine 10 (H3S10ph) during infection, suggesting a link between H3S10 phosphorylation and HSV-1 IE transcription. Interestingly, the aurora kinase inhibitors also reduced viral yields of a poxvirus (vaccinia virus) and a coronavirus (mouse hepatitis virus), demonstrating a broader antiviral effect.

## RESULTS

### Identification of four aurora kinase inhibitors against HSV-1.

Our high-throughput assay identified a subgroup of aurora kinase inhibitors that potently reduced the signal of our HSV-1 reporter virus, KOS6β (31). Here, we characterize the effects of aurora kinase inhibitors on HSV-1-infected fibroblast and epithelial cells, as well as observed effects of inhibitors on other DNA and RNA viruses. As described in Table 1, TAK 901, JNJ 7706621, PF 03814735, and AT 9283 aurora kinase inhibitors were identified as potential hits against HSV-1. TAK 901, an azacarboline, has been demonstrated to have tight-binding inhibition on aurora B kinase, with a 50% inhibitory concentration (IC_50_) of 15 nM (38). JNJ 7706621 is a dual inhibitor of aurora kinases and CDKs (39). PF 03814735 is an ATP competitive and reversible aurora kinase inhibitor. Although PF 03814735 has minimal effects on other kinases, its predominant effect in cellular assays is inhibition of aurora kinases, with an IC_50_ in the nanomolar range (40, 41). AT 9283 inhibits several kinases, including aurora kinase A and B, janus kinase, and abl kinases (42). We focused our studies on TAK 901 (TAK), JNJ 7706621 (JNJ), and PF 03814735 (PF) due to their preferential inhibition of aurora kinases.

**TABLE 1:**
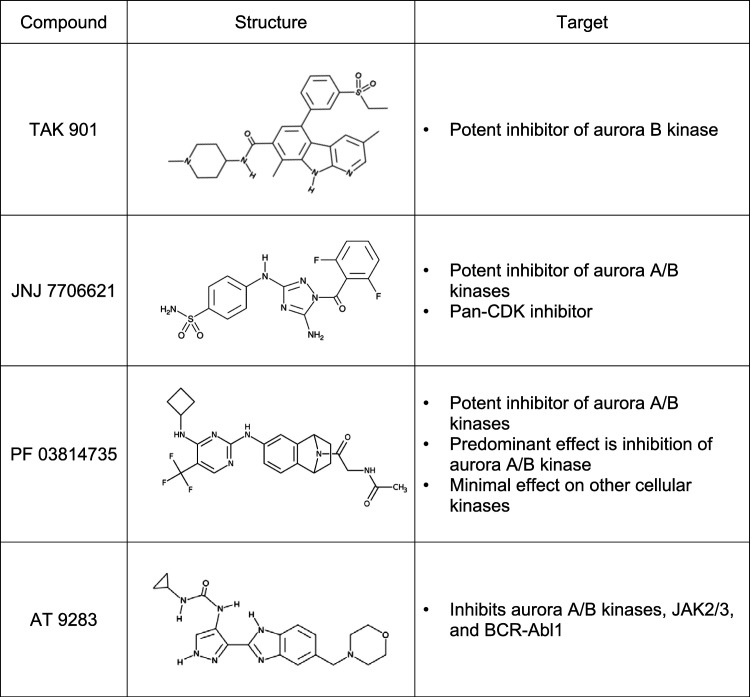
Aurora kinase inhibitor hits against HSV-1

### Treatment of HepaRG and HFF cells with aurora kinase inhibitors TAK, JNJ, and PF resulted in significant reductions in HSV-1 titers.

**FIG 1:**
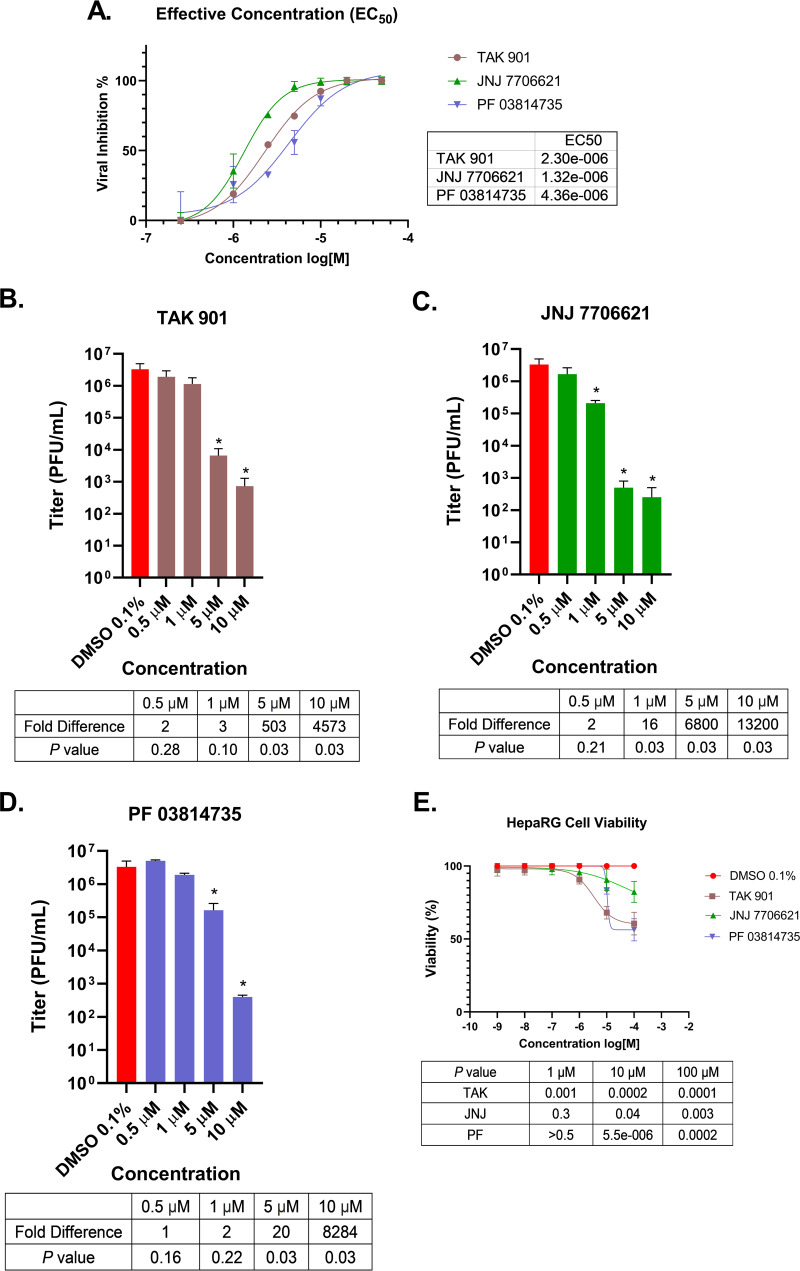
Aurora kinase inhibitors significantly reduced HSV-1 replication. (A) The effective concentration (EC_50_) for each inhibitor was determined on HepaRG cells. Viral inhibition represents the β-galactosidase signal change in HSV-1 reporter virus, KOS6β, compared to that in DMSO-treated cells. (B to D) Efficacies of TAK 901 (B), JNJ 7706621 (C), and PF 03814735 (D) on viral replication were determined by HSV-1 viral yield assays. HepaRG cells were pretreated in the presence of an aurora kinase inhibitor concentration or the DMSO vehicle. After 30 min, cells were infected with WT HSV-1 strain KOS at an MOI of 1. Viral samples were harvested at 24 hpi, and titers were determined by plaque assays. (E) alamarBlue assays were carried out to determine HepaRG cell viability during inhibitor treatment. Cells were incubated in the presence of an inhibitor at concentrations between 100 and 0.001 μM for 24 h. Error bars represent standard deviations of the means from three independent experiments. *, *P < *0.05 compared to DMSO control (Student's *t* test).

First, we determined the 50% effective concentration (EC_50_) of the aurora kinase inhibitors against HSV-1 reporter virus KOS6β. The EC_50_ values were 2.30 μM, 1.32 μM, and 4.36 μM for TAK, JNJ, and PF, respectively (Fig. 1A). Then, we assessed impacts of the aurora kinase inhibitors on virus production by performing viral yield assays. HepaRG cells are a human hepatocyte cell line that is permissive for HSV-1 replication, and they are a useful cell model for drug studies, due to their metabolic profile (43). HepaRG cells were pretreated with each aurora kinase inhibitor at 10 μM, 5 μM, 1 μM, and 0.5 μM, or the vehicle (dimethyl sulfoxide [DMSO]). After 30 min of pretreatment, cells were infected with HSV-1 strain KOS at a multiplicity of infection (MOI) of 1 PFU/cell. Twenty-four hours postinfection (hpi), viral titers were analyzed. All three compounds exhibited dose-dependent reductions in viral yields compared to the control. Treatment with TAK reduced viral yields 4,573- and 503-fold at 10 and 5 μM, respectively (Fig. 1B). JNJ treatment reduced viral yields 13,200-, 6,800-, and 16-fold at 10, 5, and 1 μM, respectively (Fig. 1C). Finally, PF treatment reduced viral yields 8,284- and 20-fold at 10 and 5 μM, respectively (Fig. 1D). These reductions in viral yields for the 3 inhibitors at 10 μM were similar to reductions observed for a known inhibitor of HSV-1 and CDKs, roscovitine (C. Y. Ly and D. J. Davido, unpublished data) (21). Cytotoxicity of the compounds in HepaRG cells was evaluated using an alamarBlue assay, and cell viability was determined using concentrations between 100 and 0.001 μM (Fig. 1E). TAK treatments at 100 μM, 10 μM, and 1 μM resulted in cell viabilities of 51%, 64%, and 96%, respectively. JNJ displayed the least cytotoxic effects of all compounds, with 82% viability at 100 μM, 91% viability at 10 μM, and 96% viability 1 μM. PF had lower cell viability (56%) at 100 μM, but cell viability increased at 10 μM (83%) and 1 μM (100%). Overall, cell viabilities were notably reduced for all 3 inhibitors at 10 μM and 100 μM but only for TAK at 1 μM. Because of these limited-to-moderate effects on cell viability, we continued to use 10 μM concentrations in subsequent experiments.

**FIG 2:**
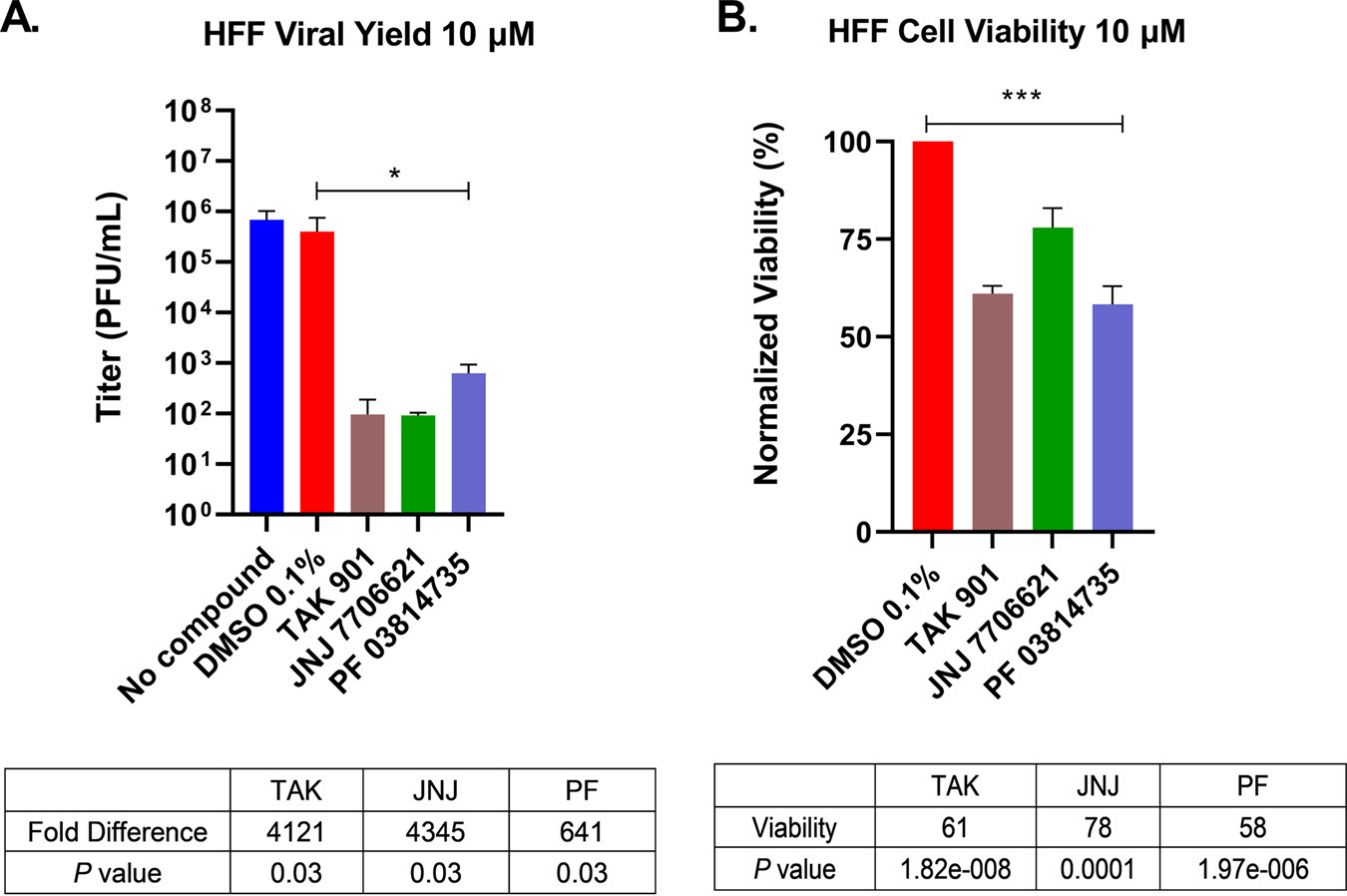
Aurora kinase inhibitors diminished HSV-1 titers in primary cultures of HFFs. (A) HFF cells were pretreated with an aurora kinase inhibitor at 10 μM for 30 min. Cells were then infected with HSV-1 strain KOS at an MOI of 1. Virus-infected samples were harvested at 24 hpi, and titers were determined by standard plaque assays. (B) alamarBlue assays measured cell viability of HFF cells after 24 h of treatment at 10 μM concentration for each kinase inhibitor. Data represent the means and standard deviations of three independent experiments. *, *P < *0.05; ***, *P ≤ *0.0001 relative to DMSO control (Student's *t* test).

Although continuous cell lines are robust and easier to work with, they are modified to be propagated indefinitely. As aurora kinases are important for cell cycle progression, we sought to test the efficacy of these inhibitors in a primary cell line, human foreskin fibroblasts (HFFs). HFF cells are permissive to HSV-1 and retain their physiological *in vivo* state due to their finite life span. Treating HFFs with each compound at 10 μM resulted in substantially lower viral yields compared to the DMSO control during HSV-1 infection (Fig. 2A). TAK and JNJ reduced viral yields ~4,000-fold, whereas treatment with PF reduced viral yields ~640-fold. We noticed that these inhibitors did show statistical differences in HFF cell viabilities, but they were unlikely to account for the ~2- to 3-log reduction in viral yields (Fig. 2B). Overall, these results indicated that the aurora kinase inhibitors can reduce HSV-1 replication in two different human cell types.

### Aurora kinase inhibitors are not virucidal against HSV-1 and are effective early in viral infection.

**FIG 3:**
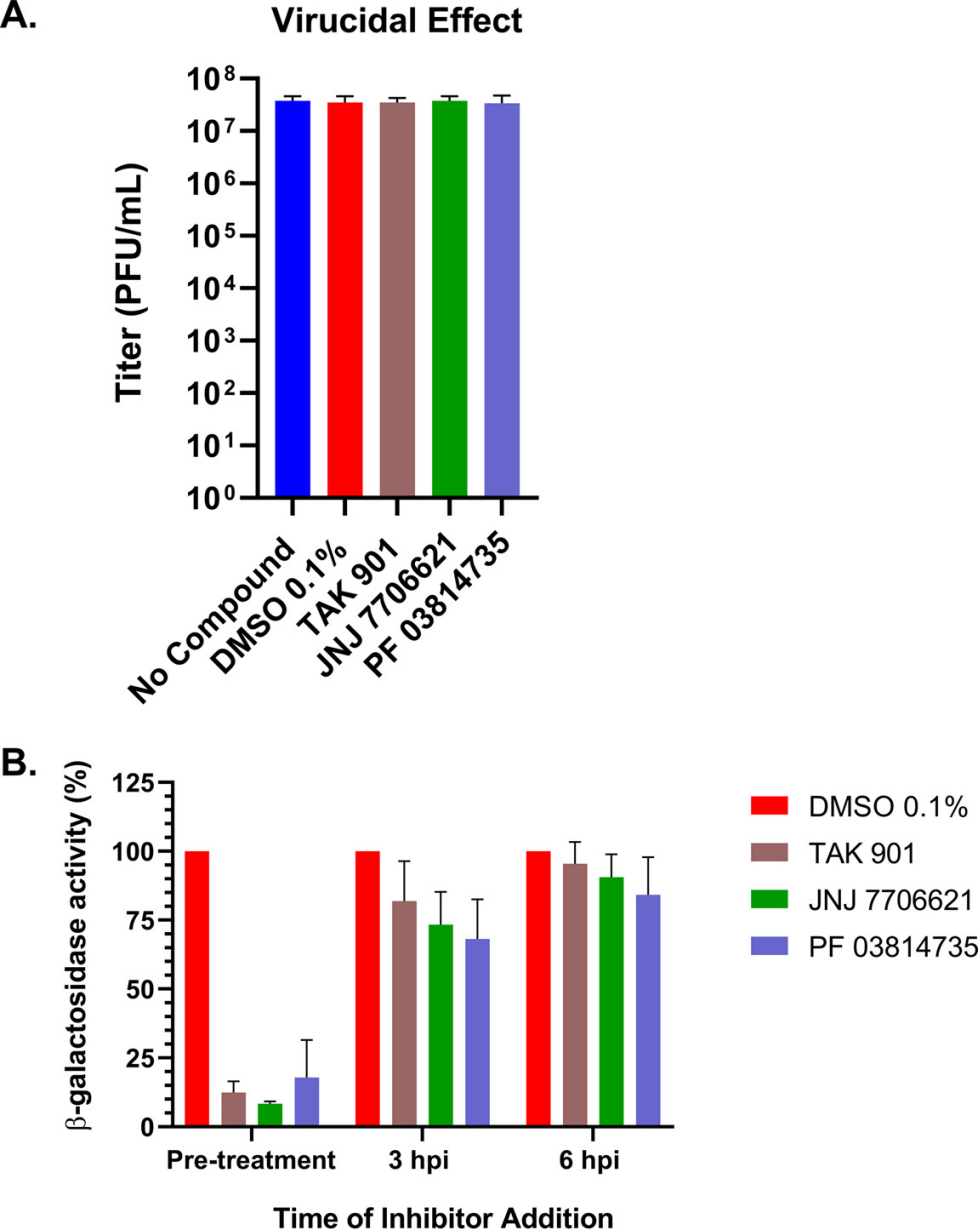
Aurora kinase inhibitors did not affect HSV-1 virions and inhibited an HSV-1 promoter during pretreatment of HSV-1 viral infection. (A) HSV-1 strain KOS viral preparations were directly treated with each kinase inhibitor (10 μM) in medium for 3 h at 37°C. Samples were diluted 10-fold in medium, and viral titers were determined by plaque assay. (B) Aurora kinase inhibitors (10 μM) were added at pretreatment, 30 min prior to infection, and at 3 hpi and 6 hpi with KOS6β, which contained an ICP6 promoter:*lacZ* expression cassette. At 24 hpi, β-galactosidase activities were measured. Error bars represent standard deviations of the means from three independent experiments performed in duplicate.

To determine if the aurora kinase inhibitors had a direct effect on HSV-1 infectious virions, we incubated HSV-1 KOS with 10 μM concentrations of the corresponding aurora kinase inhibitor. After 3 h of incubation, viral titers were determined by standard plaque assays. Compared to the vehicle, there was no change in viral titers upon aurora kinase inhibitor treatment (Fig. 3A). This experiment demonstrated that the aurora kinase inhibitors did not directly impact HSV-1 virions, indicating that the reduction in viral titers we observed with these inhibitors was from restricting productive infection.

Our high-throughput screen with the HSV-1 β-galactosidase reporter virus, KOS6β, showed that TAK, JNJ, and PF blocked ICP6 promoter activity, which is dependent on ICP0. To identify the time frame of this impairment, cells were treated with aurora kinase inhibitors at different times during KOS6β infection, and β-galactosidase assays were performed. Our data indicated that viral gene expression was diminished within the first 3 h of infection after pretreatment or inhibitor treatment prior to infection (Fig. 3B). The addition of TAK, JNJ, or PF at 3 and 6 hpi, however, largely negated their inhibitory effects (Fig. 3B). These results suggested that the aurora kinase inhibitors impair an early step of the HSV-1 lytic cycle.

### Aurora kinase inhibitors reduce HSV-1 IE transcript levels and diminish viral protein expression.

**FIG 4:**
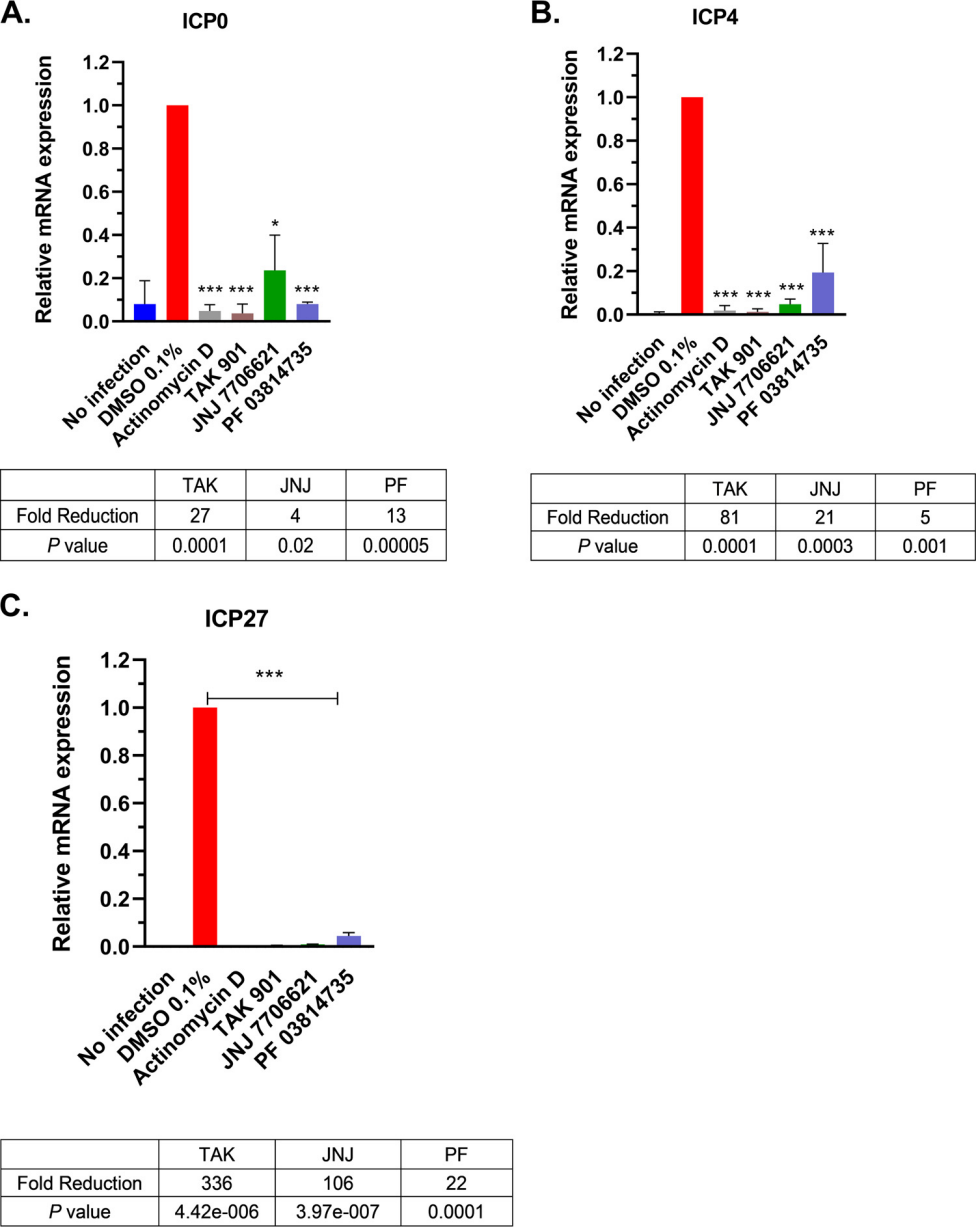
HSV-1 IE transcripts were reduced in the presence of aurora kinase inhibitors. HSV-1 IE transcript levels were analyzed by RT-qPCR for ICP0 (A), ICP4 (B), and ICP27 (C). HepaRG cells were pretreated for 30 min with TAK, JNJ, PF, or DMSO (vehicle). Positive control for the inhibition of transcription was actinomycin D (2 μg/mL). Cells were infected with HSV-1 strain KOS, and RNA was isolated from samples at 4 hpi. cDNAs from infected cells were analyzed by qPCR for ICP0, ICP4, and ICP27 and normalized to the housekeeping gene, 18S rRNA. The table below each graph is the relative mean fold decrease of each inhibitor treatment relative to the DMSO control. Error bars represent the standard deviations of two independent experiment performed in triplicate. *, *P < *0.05; ***, *P ≤ *0.001 versus DMSO control (Student's *t* test).

We next assessed the effects of the aurora kinase inhibitors on HSV-1 IE mRNA levels. We performed reverse transcription-quantitative PCR (RT-qPCR) to measure the transcript levels of ICP0, ICP4, and ICP27 in the absence or presence of TAK, JNJ, or PF, and we utilized actinomycin D, an RNA polymerase inhibitor, as a positive control. HepaRG cells were pretreated for 30 min with either DMSO, one of the kinase inhibitors at 10 μM, or actinomycin D (2 μg/mL). At 4 hpi, total RNA was isolated, and IE transcripts were reverse transcribed to cDNA. The relative IE mRNA expression was calculated using the comparative threshold cycle method (ΔΔ*C_T_*) and then compared to DMSO (0.1%) levels. ICP0 is an important transactivator of downstream genes in HSV-1 and is involved in helping HSV-1 evade the host immune response (44). ICP0 transcript levels showed significant decreases upon aurora kinase inhibitor treatment, from 4- to 27-fold compared to the control (Fig. 4A). ICP4 mRNA levels were prominently reduced 5- to 81-fold for all three kinase inhibitor treatments relative to DMSO (Fig. 4B). ICP4 is an essential IE viral protein involved in recruiting the host transcription machinery to viral promoters to activate early and late viral genes (45). The ICP27 mRNA levels showed the greatest reductions, of 22- to 336-fold (Fig. 4C). ICP27 has several important activities in HSV-1 lytic infection, including the activation of late genes and modulation of host mRNA biogenesis (46, 47). Thus, inhibition of aurora kinases appears to significantly impair IE transcription. As infected cultures were treated for a total of 4.5 h, it is highly unlikely that the prominent reductions in IE transcript levels were due to any cytotoxic effects caused by the kinase inhibitors.

**FIG 5:**
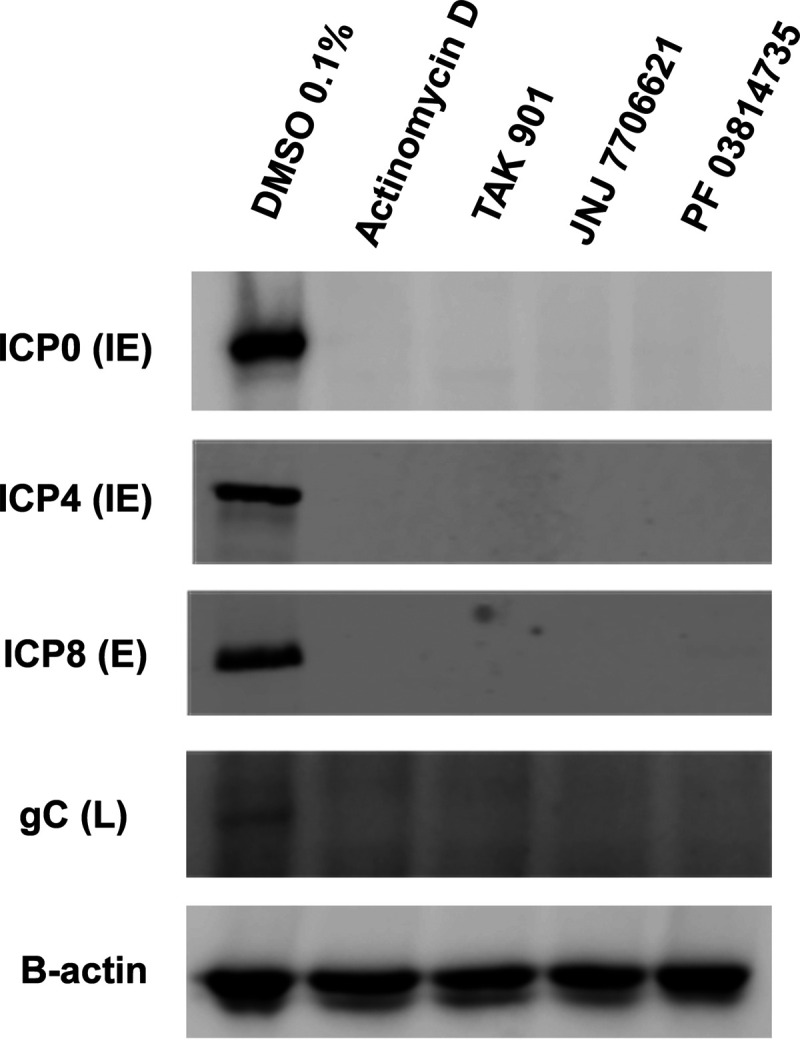
Aurora kinase inhibitors decreased HSV-1 protein expression. HepaRG cells were pretreated for 30 min with DMSO, actinomycin D (2 μg/mL), TAK, JNJ, or PF at 10 μM. Cells were infected with HSV-1 strain KOS at an MOI of 2, and cell lysates were collected at 6 hpi. Western blots were probed for viral proteins of ICP0, ICP4, ICP8, and gC, and β-actin was used as a loading control.

The significant decrease in IE transcription indicated that HSV-1 protein expression is likely impaired. To investigate this possibility, we examined HSV-1 protein expression from all three viral gene classes by Western blotting (Fig. 5). Specifically, we probed for ICP0 and ICP4 from the IE class, ICP8 from the early (E) class, and glycoprotein C (gC) from the late (L) class. HepaRG cells were pretreated with 10 μM TAK, JNJ, PF, or actinomycin D, and cells were infected with HSV-1 strain KOS and collected at 6 hpi. All HSV-1 protein levels were diminished in the presence of the aurora kinase inhibitors and actinomycin D, in contrast to the DMSO control. Taken together, these results confirmed the decreases in HSV-1 transcription (Fig. 4) resulted in the loss of viral protein expression for all classes of HSV-1 genes.

### Aurora kinase inhibitors potently diminished the phosphorylation of histone H3 at serine 10 residue.

**FIG 6:**
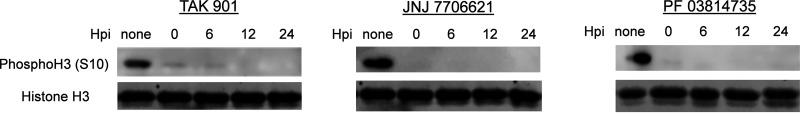
Aurora kinase inhibitors inhibited phosphorylation of H3 at serine 10 during HSV-1 lytic infection. HepaRG cells were pretreated with each kinase inhibitor or mock treated with 0.1% DMSO (none) and harvested from 0 to 24 hpi. Protein levels were detected by Western blotting with an anti-histone H3 serine 10 phosphorylation [PhosphoH3 (S10)] or anti-histone H3 antibody.

Aurora B kinase is critical for the phosphorylation of histone H3 at the serine 10 residue (H3S10ph) (33). It has been reported that TAK, JNJ, and PF prevent H3S10ph (38–41). To test whether the aurora kinase inhibitors suppressed H3S10ph during HSV-1 infection, HepaRG cells were pretreated with each aurora kinase inhibitor, and cells were then infected with strain KOS. Samples were harvested at 0, 6, 12, and 24 hpi. Interestingly, the levels of H3S10ph during HSV-1 infection were drastically reduced in the presence of the aurora kinase inhibitors compared to no treatment (Fig. 6), while levels of histone H3 levels during infection remained stable. This experiment validated that the aurora kinase inhibitors restrict the phosphorylation of a known target. Kulej et al. demonstrated in a global proteome study that H3S10ph was increased after HSV-1 infection and peaked at 9 hpi. They also determined the most abundant posttranslational modifications during HSV-1 lytic infection were histone H3 trimethylated lysine 9 and phosphorylated serine 10 (H3K9me3S10ph) (48). These data suggest that H3S10ph may be an important modification for regulating HSV-1 lytic infection, potentially counteracting the repressive effects of H3K9me3. Given that the aurora kinase inhibitors block H3S10ph, this outcome provides potential insight into the role of aurora kinase activities during HSV-1 lytic infection.

### Aurora kinase inhibitors significantly reduced viral titers of murine coronavirus and vaccinia virus but not vesicular stomatitis virus.

To investigate whether aurora kinase inhibitors altered the replication of other classes of viruses, we examined the effects of these inhibitors on murine hepatitis virus (MHV; also known as murine coronavirus), which is a positive-strand RNA virus, vaccinia virus, a DNA virus that replicates in the cytoplasm, and vesicular stomatitis virus (VSV), a negative-strand RNA virus.

**FIG 7:**
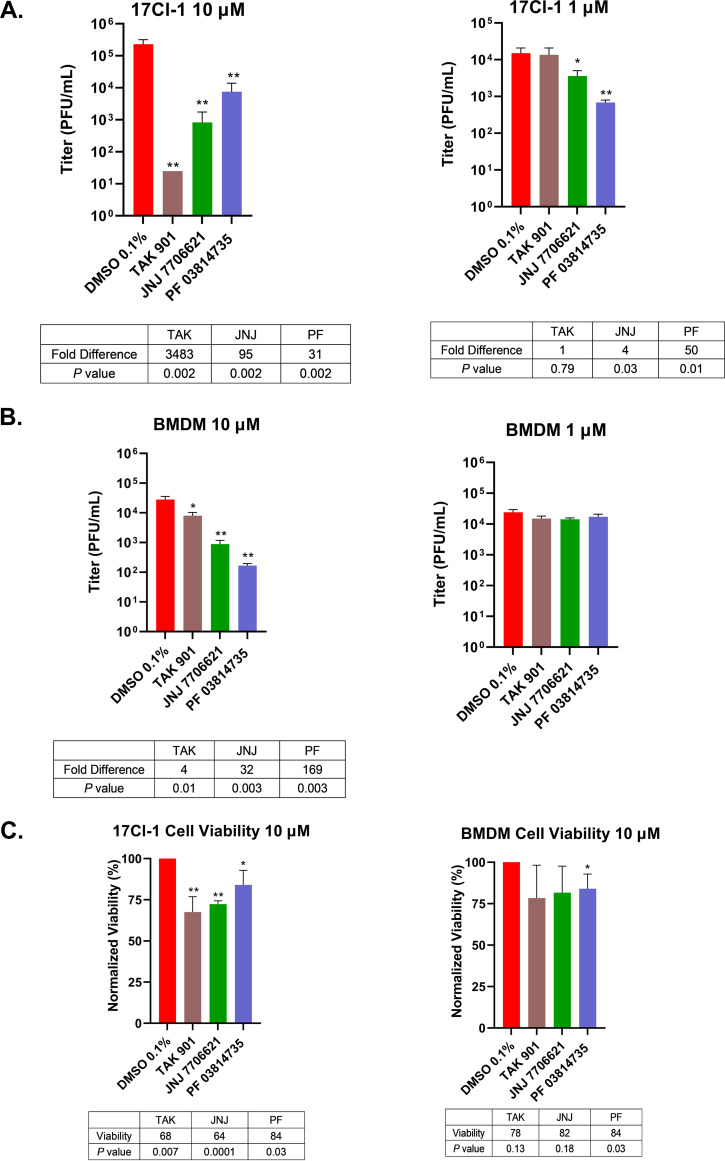
Aurora kinase inhibitors negatively impacted murine coronavirus replication. (A and B) 17Cl-1 (A) or BMDM (B) cells was pretreated with 10 μM or 1 μM of each aurora kinase inhibitor for 30 min. Cells were then infected with MHV at an MOI of 0.1 PFU/cell. Infected cells and supernatants were harvested at 18 hpi, and titers were determined by standard plaque assays. (C) The cell viability of 17Cl-1 or BMDM cells following 24 h treatment at 10 μM of each inhibitor was determined using an MTT assay. Each figure is a representative graph from three independent experiments. Error bars represent the standard deviations of the means. *, *P < *0.05; **, *P ≤ *0.01 relative to DMSO control (Student's *t* test).

A recent antiviral drug screen of kinases identified aurora kinase inhibitors as potent inhibitors of severe acute respiratory syndrome coronavirus 2 (SARS-CoV-2) replication (49). In the screen, the aurora kinase inhibitors ZM 447439 and hesperadin reduced viral cytopathic effects of SARS-CoV-2-infected cells. Based on these results, we sought to test whether TAK, JNJ, and PF impaired another coronavirus, MHV. A murine fibroblast cell line, 17Cl-1, and primary murine bone marrow-derived macrophages (BMDMs) were pretreated with aurora kinase inhibitor at 10 μM or 1 μM and then infected with MHV for 18 h. Infected cells and supernatants were collected to determine viral yields. We observed significant reductions of viral titers in aurora kinase inhibitor-treated cells for both 17Cl-1 and BMDM cells at 10 μM, with reductions ranging from 3- to 3,480-fold. JNJ and PF also significantly inhibited MHV at 1 μM in 17Cl-1 cells, though they did not reduce MHV replication in BMDMs at this concentration (Fig. 7A and B). These compounds were associated with minimal to statistically discernible decreases in cell viabilities for both cell types (Fig. 7C). These results indicated that the aurora kinase inhibitors TAK, JNJ, and PF reduce murine coronavirus replication.

**FIG 8:**
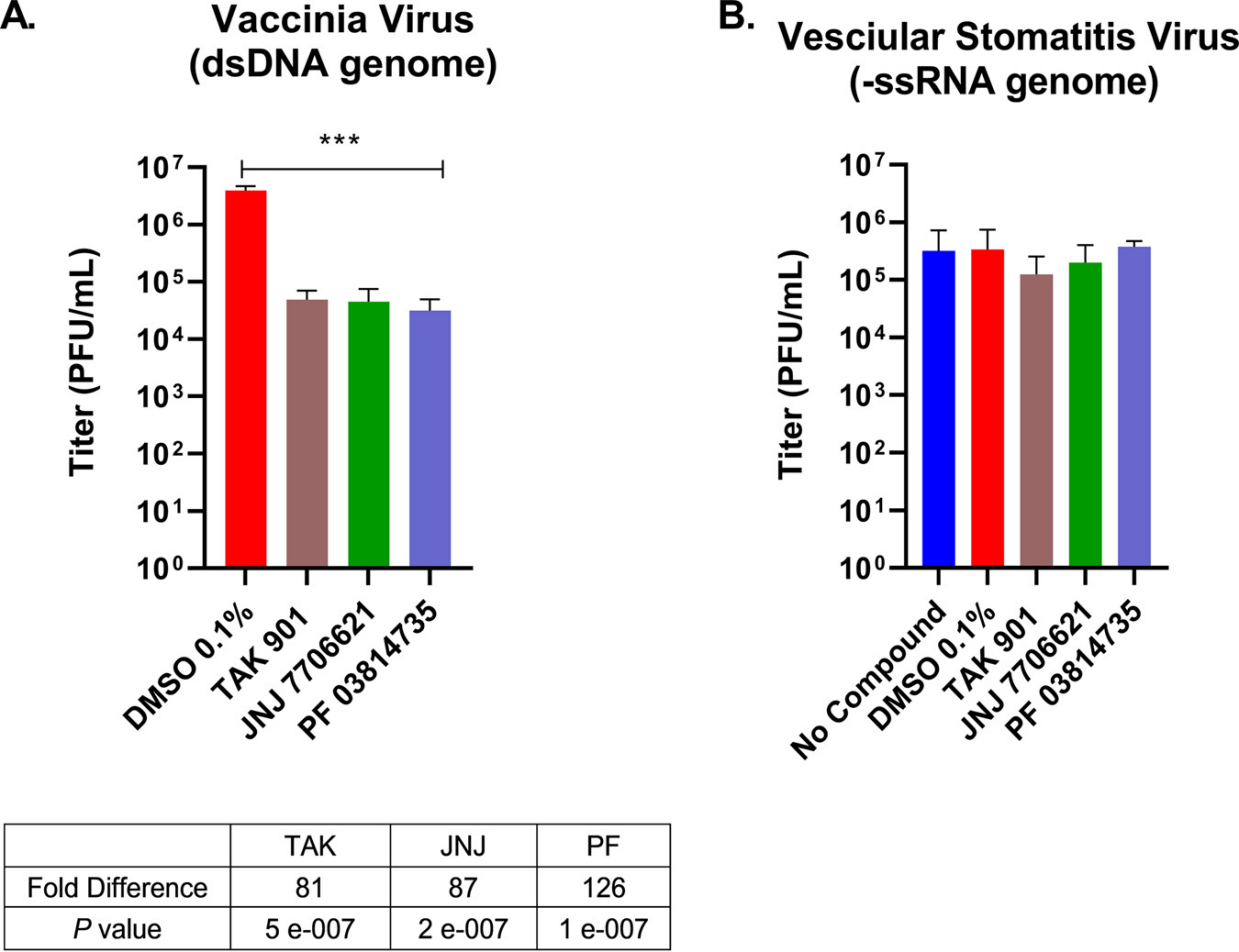
Aurora kinase inhibitors significantly reduced VACV titers but not VSV titers. (A) HFF cells were pretreated with aurora kinase inhibitor at 10 μM for 30 min. HFFs were then infected with WT VACV at an MOI of 2. VACV titers were measured 24 hpi, and titers were determined by standard plaque assays. (B) HepaRG cells were pretreated with aurora kinase inhibitor for 30 min and then infected with VSV at an MOI of 1 for 24 h. Viral samples were collected 24 hpi, and titers were determined by standard plaque assays. Error bars represent the standard deviations of the means from three independent experiments. ***, *P < *0.0001 compared with DMSO control (Student's *t* test).

We tested the aurora kinase inhibitors on vaccinia virus (VACV), a dsDNA virus that replicates within the cytoplasm (50). HFF cells were pretreated with each aurora kinase inhibitor. Cells were infected with VACV for 24 hpi, and viral yields were determined by plaque assays. We observed reductions in VACV yields with 10 μM treatments of aurora kinase inhibitors. TAK, JNJ, and PF exhibited reductions from 81- to 126-fold in VACV titers (Fig. 8A). There was no significant decrease in VACV titers at 1 μM treatment (A. Pant and Z. Yang, unpublished data). These experiments demonstrated that aurora kinase inhibitors significantly diminished VACV lytic replication.

Finally, we examined how the aurora kinase inhibitors affected vesicular stomatitis virus (VSV), a minus-sense single-stranded RNA (ssRNA) virus from the *Rhabdoviridae* family. HepaRG cells were pretreated with TAK, JNJ, or PF, and cells were then infected with VSV. Viral samples were collected 24 hpi to determine their yields. As shown in Fig. 8B, VSV-infected cells treated with aurora kinase inhibitors were able to replicate to the same levels as the DMSO control. This set of data indicates that aurora kinase inhibitors do not negatively impact VSV replication and suggest that the activities of these kinases are not required for the productive infection of all viruses.

Taken together, our results strongly suggest that the activities of aurora kinase are important in the productive infection of several viruses (i.e., HSV-1, MHV, VACV) and highlight the broad antiviral effects of aurora kinase inhibitors on viruses with diverse life cycles.

## DISCUSSION

Aurora kinases activities are important for the replication of a variety of diverse viruses (35–37). These studies demonstrate, for the first time, a potential role for aurora kinases in the HSV-1 lytic life cycle. Our experiments show the robust effect of aurora kinase inhibitors TAK, JNJ, and PF in reducing HSV-1 viral replication. Upon 10 μM aurora kinase inhibitor treatment, we observed significant reductions in HSV-1 replication in two different cell lines (Fig. 1 and 2), and these reductions were linked to major reductions in ICP0, ICP4, and ICP27 transcript levels (Fig. 4). These transcripts are important for the activation of the HSV-1 gene cascade, recruiting host machinery, and modulating cellular environment during HSV-1 infection. These decreases further abrogated viral protein expression for all HSV-1 gene classes (Fig. 5). Given the specificity of TAK as a kinase inhibitor, we propose that aurora kinase B plays a principal role in stimulating IE gene expression. Furthermore, as inhibition of viral gene expression with these compounds occurs during the early phase of viral infection (≤3 hpi, Fig. 3B), it appears that this phenotype is not directly associated with diminished cell viability and proliferation. The cumulative effects of the aurora kinases inhibitors highlighted that aurora kinase functions are utilized during an early phase of HSV-1 to promote viral replication.

While the specific mechanism of aurora kinases during lytic infection remains unknown, we hypothesize that the aurora kinase role of phosphorylating H3 on serine 10 position may be critical during HSV-1 lytic infection. Aurora B kinase is known to mediate methyl-phospho switches on H3 to alleviate transcriptional repression of heterochromatin protein 1 (HP1) (51). A methyl-phospho switch on histone H3 is when the phosphorylation of serine 10 occurs neighboring a trimethylated lysine 9 (H3K9me3/S10ph). It has been reported that H3K9me3/S10ph is the most abundant posttranslational modification during HSV-1 lytic infection, but the specific kinases mediating the methyl-phospho switches during lytic infection remain unknown (48). A previous study reported JNK mediates a methyl-phospho switch on H3 (H3K9me3/S10ph) during HSV-1 reactivation. The methyl-phospho switch stimulates viral gene expression from repressed viral heterochromatin during the early phase of HSV-1 reactivation (24). Here, we report a significant and robust inhibition of phosphorylation of H3 at serine 10 with only 30 min of pretreatment during an HSV-1 infection (Fig. 6). Future studies will examine if one or more of the aurora kinases can mediate a methyl-phospho switch on H3 to reverse HSV-1 gene silencing during lytic infection. It is also possible that the impairment of aurora kinase activities may more broadly affect mRNA synthesis or factors that play a role in HSV-1 IE gene expression, such as the transcriptional regulators Oct-1 and HCF, but this has yet to be determined.

Our studies also demonstrated the broad antiviral effects of aurora kinase inhibitors on MHV and VACV. With coronavirus infections, the data demonstrated significant reductions in viral replication after treatment with TAK, JNJ, and PF (Fig. 7). Coronavirus has a positive-sense RNA genome, with which it can be directly translated and processed in the cytoplasm after cellular entry (52, 53), and thus the mechanisms used by aurora kinases to stimulate MHV replication are likely to be significantly different from those for HSV-1. For VACV infections, there was a significant reduction in viral titers upon treatment with 10 μM TAK, JNJ, or PF (Fig. 8A). Interestingly, other aurora kinase inhibitors were also been found to suppress VACV replication in a published screen identifying inhibitors of VACV (54). VACV is unusual among DNA viruses, as its replication occurs in the cytoplasm (50). Although having significant reductions in VACV titers, these reductions peaked at ~120-fold, whereas they were at least 1,000-fold for HSV-1. These observations suggest that the mechanisms of inhibition are conceivably associated with the location of viral replication.

Aurora kinases are overexpressed in a wide range of human cancers, such as various leukemias. Second-generation aurora kinase inhibitors are continuously being enhanced for selectivity (55). TAK, JNJ, and PF have been well-characterized in studies against cancer but understudied for viral diseases. Overall, our data suggest that aurora kinases have diverse roles and mechanisms for enhancing the replication of several different classes of viruses. These findings indicate that aurora kinases may be applicable as broad-spectrum antiviral targets and thus warrant further investigation.

## MATERIALS AND METHODS

### Cell, viruses, and compounds.

HepaRG cells are a human epithelial hepatocyte line derived from a liver tumor patient, a gift from Roger Everett. HepaRG cells were grown in William's E medium containing 10% fetal bovine serum (FBS), 2 mM l-glutamine, 10 U/mL penicillin, 10 U/mL streptomycin, 50 μg/mL insulin, and 50 μM hydrocortisone. Primary human foreskin fibroblasts (HFFs; a gift from Nicholas Wallace) were grown in Dulbecco’s minimal essential medium (DMEM) containing 10% FBS, 2 mM l-glutamine, 10 U/mL penicillin, and 10 U/mL streptomycin. Vero cells were cultured in DMEM as described for HFF cells. HSV-1 strain KOS (provided by Priscilla Schafer) was used in this study as wild-type HSV-1.

BS-C-1 cells, an African green monkey kidney cell line, were cultured in Eagle’s minimal essential medium (EMEM) with supplements as described above for HFF cells. All the cell lines were maintained in a humidified incubator at 37°C in 5% CO_2_. VACV Western Reserve strain was used in this study (ATCC VR-1345).

17Cl-1 cells, a murine fibroblast cell line, were cultured in enriched DMEM with 10% FBS, 100 U/mL penicillin, 100 mg/mL streptomycin, HEPES, sodium pyruvate, nonessential amino acids, and l-glutamine. Bone marrow-derived macrophages (BMDMs) isolated from wild-type (WT) mice (Jackson Laboratories) were differentiated by incubating cells in Roswell Park Memorial Institute (RPMI) medium containing 10% L929 cell supernatants, 10% FBS, sodium pyruvate, 100 U/mL penicillin, 100 mg/mL streptomycin, and l-glutamine for 7 days. HeLa cells were grown in DMEM supplemented with 10% FBS, 100 U/mL penicillin, 100 U/mL streptomycin, HEPES, sodium pyruvate, nonessential amino acids, and l-glutamine. Murine hepatitis virus strain JHM, which contains green fluorescent protein (GFP) in place of ORF4, was used in this study (53). Vesicular stomatitis virus (VSV-eGFP), which contains enhanced green fluorescent protein between the G and L genes, was a gift from Asit Pattnaik. TAK 901, JNJ 7706621, and PF 03814735 were purchased from Selleck Chemistry or MilliporeSigma.

### Viral yields.

For HSV-1 viral yields, HepaRG cells (1 × 10^5^) were seeded in a 12-well plate in William’s E medium. Each well was pretreated for 30 min with a kinase inhibitor (10 μM, 5 μM, 1 μM, or 0.5 μM) or DMSO at 0.1%. HFF cells were (1 × 10^5^) were seeded in a 12-well plate in DMEM complete medium, and each well was pretreated with 10 μM kinase inhibitor. Each well was infected with HSV-1 strain KOS at an MOI of 1 and incubated for 1 h. At 1 hpi, wells were washed twice with PBS (plus each inhibitor) to synchronize infections. After 24 hpi, cells were harvested into supernatants and collectively used to determine viral titers by standard plaque assays on Vero cells (56). Viral yields for VSV were performed in HepaRG cells at a 10 μM concentration of each kinase inhibitor as described above, and viral titers were determined by standard plaque assays on Vero cells.

To calculate the viral yields of VACV, HFFs were cultured in DMEM in 12-well plates. Cells were pretreated in the presence of DMSO or 10 μM of an aurora kinase inhibitor for 30 min and then infected with VACV at an MOI of 2. After 24 hpi, the viral titers were measured by plaque assays on B-SC-1 monolayers as described previously (57).

For MHV viral yield assays, 17Cl-1 (3 × 10^4^) or BMDM (8 × 10^4^) cells were seeded per well in a 24-well plate. Each well was pretreated for 30 min with DMSO at 0.1% or TAK 901, JNJ 7706621, or PF 03814735 at 10 μM or 1 μM. Cells were then infected with MHV strain JHM at an MOI of 0.1 for 1 h, and viral inoculum was removed and replaced with medium containing the inhibitor. Samples (cells and media) were harvested at 18 hpi, and viral titers were determined by standard plaque assays on HeLa cells.

### Mice.

Pathogen-free C57BL/6 (B6) mice were purchased from Jackson Laboratories and cared for according to the *Guide for the Care and Use of Laboratory Animals* (58).

### Ethics statement.

The protocol for using the B6 mice was approved by the University of Kansas Institutional Animal Care and Use Committee (IACUC).

### Cell viability assays.

HepaRG and HFF cell viabilities were measured using an alamarBlue assay (Bio-Rad) following the manufacturer’s protocol. 17Cl-1 cell and BMDM viabilities were measured using the CyQUANT MTT cell proliferation assay (Thermo Fisher Scientific) following the manufacturer’s protocol. Means and standard deviations were calculated from at least three independent experiments.

### Virucidal effect.

Using a 12-well plate, HSV-1 strain KOS viral stocks containing 3 × 10^7^ PFU were directly treated with each kinase inhibitor (10 μM) in William’s E medium for 3 h at 37°C. Samples were diluted 10-fold in DMEM complete, and viral titers were determined by standard plaque assays. The averages and standard deviations were determined for three independent experiments.

### Time course addition.

HepaRG cells (1 × 10^4^) were seeded in a 96-well plate in William’s E medium and incubated overnight. Cells were infected with HSV-1 recombinant KOS6β at an MOI of 5, and the aurora kinase inhibitors (10 μM) were added at respective time points during infection: pretreatment (30 min prior to infection), 3 hpi, and 6 hpi. At 24 hpi, samples were harvested in 1% IGEPAL lysis buffer (IGEPAL CA-630; Thermo Fisher Scientific), and cell lysates were processed in a 1× CPRG solution (10X CPRG solution: 80 mM chlorophenol red-β-d-galactopyranoside, 0.6 M NaHPO_4_, 0.4 M NaH_2_PO_4_, 0.1 M KCl, 0.01 M MgSO_4_, 0.5 M 2-mercaptoethanol). Absorbance was measured at 595 nm 1 h post-addition of CPRG solution using a spectrophotometer (BioTEK Synergy, BioTek Instruments). The means and standard deviations for samples were determined from three independent experiments.

### Viral transcript assay and RT-qPCR.

HepaRG cells (2.5 × 10^5^) was seeded in a 12-well plate in William’s E medium. Cells were pretreated for 30 min with each kinase inhibitor (10 μM), DMSO at 0.1%, or actinomycin D (2 μg/mL) at 37°C. Each well was infected with HSV-1 strain KOS at an MOI of 2 and incubated at 37°C for 4 h. At 4 hpi, cells were harvested in TRIzol (Invitrogen) and frozen at −80°C overnight. Samples were then mixed twice with chloroform and centrifuged, and the aqueous layers were isolated. Glycogen (RNA grade; Thermo Fisher Scientific) was added to each sample with isopropanol. Samples were placed at −20°C for 30 min and then centrifuged at high speeds to collect RNA pellets. Pellets were washed 3 times in 75% ethanol solution and air-dried. RNAs were dissolved in molecular-grade water and treated with RQ1 DNase (Promega). RNAs were converted to cDNA with the High-Capacity cDNA reverse transcription kit (Applied Biosystems); random primers were utilized for 18S rRNA, and oligo-dT(18) primers were utilized for ICP0, ICP4, and ICP27 mRNAs. For qPCR analyses, FastStart Universal SYBR green master (Rox; MilliporeSigma) was used. Transcripts were amplified using primers for ICP0, ICP4, ICP27, and 18S rRNA sequences (Table 2). Signal was measured using an Applied Biosystems QuantStudio 3.

**TABLE 2:** Primers used for RT-qPCR

Gene	Forward primer	Reverse primer
18S	5′-CCAGTAAGTGCGGGTCATAAGC-3′	5′-GCCTCACTAAACCATCCAATCGG-3′
ICP0	5′-AGCGAGTACCCGCCGGCCTG-3′	5′-CAGGTCTCGGTCGCAGGGAAAC-3′
ICP4	5′-TGATCACGCGGCTGCTGTA-3′	5′-TGATGAAGGAGCTGCTGTTGCG-3′
ICP27	5′-GTCCAAGATGTGCATCCACCACAA-3′	5′-TGCAATGTCCTTAATGTCCGCCAG-3′

### Western blot assays.

**(i) Viral proteins.** HepaRG cells (2.5 × 10^5^) were cultured per well in a 12-well plate containing William’s E medium. Each well was pretreated for 30 min with each kinase inhibitor (10 μM) or DMSO at 0.1% at 37°C in 5% CO_2_. Each well was infected with HSV-1 strain KOS at an MOI of 2 and incubated at 37°C for 6 h. A 50-μL volume of 1× Laemmli buffer was added, and samples were heated at 95°C for 10 min. To probe ICP0, ICP4, ICP8, gC, and actin, samples were loaded into a 3 to 8% Tris-acetate gel in 1× Tris-acetate running buffer (NuPAGE, ThermoFisher Scientific). Proteins were transferred to nitrocellulose membranes (Cytiva) using a semidry transfer unit at 54 mA per membrane for 1.5 h (TE 77; Amersham Biosciences). Each membrane was blocked in 5% bovine serum albumin in Tris-buffered saline with 0.1% Tween 20 (TBS-T) overnight at 4°C. Primary antibodies ICP0 (catalog number sc-53070; diluted 1:250), ICP4 (catalog number sc-69809; diluted 1:500), ICP8 (catalog number sc-53330; diluted 1:500), gC (catalog number sc-56982; diluted 1:500), and actin (catalog number sc-47778; diluted 1:1,000) from Santa Cruz Biotechnology were incubated with membranes overnight at 4°C. Membranes were washed 3 times in TBS-T, and goat anti-mouse IgG horseradish peroxidase-conjugated secondary antibody IgG (catalog number 115-035-146; diluted 1:1,000) from Jackson ImmunoResearch was added at room temperature for 1 h. Membranes were washed 3 times in TBS-T and developed using SuperSignal West Pico chemiluminescent substrate (Thermo Fisher Scientific). Pictures were captured with a LI-COR XF imaging system and analyzed with LI-COR Image Studio.

**(ii) PhosphoH3 (Ser 10) and histone H3 analyses.** HepaRG cells (1 × 10^5^) were cultured in a 12-well plate in Williams E complete medium. Each well was pretreated for 30 min with a kinase inhibitor (10 μM) or DMSO at 0.1% at 37°C in 5% CO_2_. Each well was infected with HSV-1 strain KOS at an MOI of 2 and incubated at 37°C for 6 h. Medium was removed from each well, 50 μL of 1× Laemmli buffer was added per well, and samples were heated at 95°C for 10 min. Samples were loaded on a 10 to 12% SDS-PAGE gel in 1× SDS running buffer. Proteins were transferred to nitrocellulose, and blots were probed for PhosphoH3 (Ser 10) (catalog number 9701; diluted 1:1,000) and histone H3 (catalog number 9715; diluted 1:1,000) from Cell Signaling Technology. Membranes were washed 3 times in TBS-T, and secondary antibody goat anti-rabbit IgG (catalog number 111-035-144; diluted 1:1,000) from Jackson ImmunoResearch was added at room temperature for 1 h. Membranes were analyzed as described above for the detection of viral proteins.

### Statistical analyses.

All statistical analysis was performed using independent biological experiments. Prism 9 software (GraphPad) and Microsoft Excel were utilized for data, graphs, and statistical analyses.

## References

[1] Rauch J, Volinsky N, Romano D, Kolch W. 2011. The secret life of kinases: functions beyond catalysis. Cell Commun Signal 9:23. doi:10.1186/1478-811X-9-23. 22035226 PMC3215182

[2] Cheng H-C, Qi RZ, Paudel H, Zhu H-J. 2011. Regulation and function of protein kinases and phosphatases. Enzyme Res 2011:794089. doi:10.4061/2011/794089. 22195276 PMC3238372

[3] Kim C-H, Kim D-E, Kim D-H, Min G-H, Park J-W, Kim Y-B, Sung CK, Yim H. 2022. Mitotic protein kinase-driven crosstalk of machineries for mitosis and metastasis. Exp Mol Med 54:414–425. doi:10.1038/s12276-022-00750-y. 35379935 PMC9076678

[4] Baltussen LL, Rosianu F, Ultanir SK. 2018. Kinases in synaptic development and neurological diseases. Prog Neuropsychopharmacol Biol Psychiatry 84:343–352. doi:10.1016/j.pnpbp.2017.12.006. 29241837

[5] Panteva M, Korkaya H, Jameel S. 2003. Hepatitis viruses and the MAPK pathway: is this a survival strategy? Virus Res 92:131–140. doi:10.1016/S0168-1702(02)00356-8. 12686421

[6] Langland JO, Cameron JM, Heck MC, Jancovich JK, Jacobs BL. 2006. Inhibition of PKR by RNA and DNA viruses. Virus Res 119:100–110. doi:10.1016/j.virusres.2005.10.014. 16704884

[7] Beziau A, Brand D, Piver E. 2020. The role of phosphatidylinositol phosphate kinases during viral infection. Viruses 12:1124. doi:10.3390/v12101124. 33022924 PMC7599803

[8] Bonjardim CA. 2017. Viral exploitation of the MEK/ERK pathway: a tale of vaccinia virus and other viruses. Virology 507:267–275. doi:10.1016/j.virol.2016.12.011. 28526201

[9] Mondal A, Dawson AR, Potts GK, Freiberger EC, Baker SF, Moser LA, Bernard KA, Coon JJ, Mehle A. 2017. Influenza virus recruits host protein kinase C to control assembly and activity of its replication machinery. Elife 6:e26910. doi:10.7554/eLife.26910. 28758638 PMC5791932

[10] Meineke R, Rimmelzwaan G, Elbahesh H. 2019. Influenza virus infections and cellular kinases. Viruses 11:171. doi:10.3390/v11020171. 30791550 PMC6410056

[11] Hsiang T-Y, Zhou L, Krug RM. 2012. Roles of the phosphorylation of specific serines and threonines in the NS1 protein of human influenza A viruses. J Virol 86:10370–10376. doi:10.1128/JVI.00732-12. 22787231 PMC3457261

[12] Xie J, Zhang S, Hu Y, Li D, Cui J, Xue J, Zhang G, Khachigian LM, Wong J, Sun L, Wang M. 2014. Regulatory roles of c-Jun in H5N1 influenza virus replication and host inflammation. Biochim Biophys Acta 1842:2479–2488. doi:10.1016/j.bbadis.2014.04.017. 24780373

[13] Liou L-Y, Herrmann CH, Rice AP. 2004. Human immunodeficiency virus type 1 infection induces cyclin T1 expression in macrophages. J Virol 78:8114–8119. doi:10.1128/JVI.78.15.8114-8119.2004. 15254183 PMC446126

[14] Rice AP. 2017. The HIV-1 Tat protein: mechanism of action and target for HIV-1 cure strategies. Curr Pharm Des 23:4098–4102. doi:10.2174/1381612823666170704130635. 28677507 PMC5700838

[15] Smith MC, Bayless AM, Goddard ET, Davido DJ. 2011. CK2 inhibitors increase the sensitivity of HSV-1 to interferon-β. Antiviral Res 91:259–266. doi:10.1016/j.antiviral.2011.06.009. 21722672 PMC3159797

[16] Sciortino MT, Parisi T, Siracusano G, Mastino A, Taddeo B, Roizman B. 2013. The virion host shutoff RNase plays a key role in blocking the activation of protein kinase R in cells infected with herpes simplex virus 1. J Virol 87:3271–3276. doi:10.1128/JVI.03049-12. 23302873 PMC3592158

[17] Sciabica KS, Dai QJ, Sandri-Goldin RM. 2003. ICP27 interacts with SRPK1 to mediate HSV splicing inhibition by altering SR protein phosphorylation. EMBO J 22:1608–1619. doi:10.1093/emboj/cdg166. 12660167 PMC152910

[18] Leach NR, Roller RJ. 2010. Significance of host cell kinases in herpes simplex virus type 1 egress and lamin-associated protein disassembly from the nuclear lamina. Virology 406:127–137. doi:10.1016/j.virol.2010.07.002. 20674954 PMC2948959

[19] Yura Y, Kusaka J, Tsujimoto H, Yoshioka Y, Yoshida H, Sato M. 1997. Effects of protein tyrosine kinase inhibitors on the replication of herpes simplex virus and the phosphorylation of viral proteins. Intervirology 40:7–14. doi:10.1159/000150515. 9268765

[20] Bell C, Desjardins M, Thibault P, Radtke K. 2013. Proteomics analysis of herpes simplex virus type 1-infected cells reveals dynamic changes of viral protein expression, ubiquitylation, and phosphorylation. J Proteome Res 12:1820–1829. doi:10.1021/pr301157j. 23418649

[21] Schang LM, Phillips J, Schaffer PA. 1998. Requirement for cellular cyclin-dependent kinases in herpes simplex virus replication and transcription. J Virol 72:5626–5637. doi:10.1128/JVI.72.7.5626-5637.1998. 9621021 PMC110224

[22] Davido DJ, Von Zagorski WF, Maul GG, Schaffer PA. 2003. The differential requirement for cyclin-dependent kinase activities distinguishes two functions of herpes simplex virus type 1 ICP0. J Virol 77:12603–12616. doi:10.1128/jvi.77.23.12603-12616.2003. 14610183 PMC262587

[23] Advani SJ, Weichselbaum RR, Roizman B. 2000. The role of cdc2 in the expression of herpes simplex virus genes. Proc Natl Acad Sci USA 97:10996–11001. doi:10.1073/pnas.200375297. 10995483 PMC27137

[24] Cliffe AR, Arbuckle JH, Vogel JL, Geden MJ, Rothbart SB, Cusack CL, Strahl BD, Kristie TM, Deshmukh M. 2015. Neuronal stress pathway mediating a histone methyl/phospho switch is required for herpes simplex virus reactivation. Cell Host Microbe 18:649–658. doi:10.1016/j.chom.2015.11.007. 26651941 PMC4681005

[25] Kato A, Kawaguchi Y. 2018. Us3 protein kinase encoded by HSV: the precise function and mechanism on viral life cycle. Adv Exp Med Biol 1045:45–62. doi:10.1007/978-981-10-7230-7_3. 29896662

[26] Wang K, Ni L, Wang S, Zheng C. 2014. Herpes simplex virus 1 protein kinase US3 hyperphosphorylates p65/RelA and dampens NF-κB activation. J Virol 88:7941–7951. doi:10.1128/JVI.03394-13. 24807716 PMC4097809

[27] Duong-Ly KC, Peterson JR. 2013. The human kinome and kinase inhibition. Curr Protoc Pharmacol Chapter 2:Unit2.9. doi:10.1002/0471141755.ph0209s60. PMC4128285 23456613

[28] Roizman B, Knipe DM, Whitley RJ. 2013. Herpes simplex viruses, p 1823–1897. *In* Knipe DM, Howley PM, Griffin DE, Martin MA, Lamb RA (ed), Fields Virology. Lippincott Williams & Wilkins, Philadelphia, PA.

[29] Piret J, Boivin G. 2011. Resistance of herpes simplex viruses to nucleoside analogues: mechanisms, prevalence, and management. Antimicrob Agents Chemother 55:459–472. doi:10.1128/AAC.00615-10. 21078929 PMC3028810

[30] Rousseau A, Pharm SB, Gueudry J, Deback C, Haigh O, Schweitzer C, Boutolleau D, Labetoulle M. 2022. Acyclovir-resistant HSV-1 keratitis: a concerning and emerging clinical challenge. Am J Ophthalmol 238:110–119. doi:10.1016/j.ajo.2022.01.010. 35033543

[31] Ly CY, Yu C, McDonald PR, Roy A, Johnson DK, Davido DJ. 2021. Simple and rapid high-throughput assay to identify HSV-1 ICP0 transactivation inhibitors. Antiviral Res 194:105160. doi:10.1016/j.antiviral.2021.105160. 34384824 PMC8686521

[32] Dutertre S, Descamps S, Prigent C. 2002. On the role of aurora-A in centrosome function. Oncogene 21:6175–6183. doi:10.1038/sj.onc.1205775. 12214247

[33] Willems E, Dedobbeleer M, Digregorio M, Lombard A, Lumapat PN, Rogister B. 2018. The functional diversity of Aurora kinases: a comprehensive review. Cell Div 13:7–19. doi:10.1186/s13008-018-0040-6. 30250494 PMC6146527

[34] Vader G, Medema RH, Lens SMA. 2006. The chromosomal passenger complex: guiding Aurora-B through mitosis. J Cell Biol 173:833–837. doi:10.1083/jcb.200604032. 16769825 PMC2063908

[35] Jeong GU, Ahn B-Y. 2019. Aurora kinase A promotes hepatitis B virus replication and expression. Antiviral Res 170:104572. doi:10.1016/j.antiviral.2019.104572. 31376425

[36] Pérez-Olais JH, Ruiz-Jiménez F, Calderón-Garcia EJ, De Jesús-González LA, Hernández-Rivas R, Del Angel RM. 2019. The activity of Aurora kinase B is required for dengue virus release. Virus Res 274:197777. doi:10.1016/j.virusres.2019.197777. 31626875

[37] Jha HC, Lu J, Saha A, Cai Q, Banerjee S, Prasad MAJ, Robertson ES. 2013. EBNA3C-mediated regulation of aurora kinase B contributes to Epstein-Barr virus-induced B-cell proliferation through modulation of the activities of the retinoblastoma protein and apoptotic caspases. J Virol 87:12121–12138. doi:10.1128/JVI.02379-13. 23986604 PMC3807909

[38] Farrell P, Shi L, Matuszkiewicz J, Balakrishna D, Hoshino T, Zhang L, Elliott S, Fabrey R, Lee B, Halkowycz P, Sang B, Ishino S, Nomura T, Teratani M, Ohta Y, Grimshaw C, Paraselli B, Satou T, de Jong R. 2013. Biological characterization of TAK-901, an investigational, novel, multitargeted Aurora B kinase inhibitor. Mol Cancer Ther 12:460–470. doi:10.1158/1535-7163.MCT-12-0657. 23358665

[39] Emanuel S, Rugg CA, Gruninger RH, Lin R, Fuentes-Pesquera A, Connolly PJ, Wetter SK, Hollister B, Kruger WW, Napier C, Jolliffe L, Middleton SA. 2005. The in vitro and in vivo effects of JNJ-7706621: a dual inhibitor of cyclin-dependent kinases and aurora kinases. Cancer Res 65:9038–9046. doi:10.1158/0008-5472.CAN-05-0882. 16204078

[40] Jani JP, Arcari J, Bernardo V, Bhattacharya SK, Briere D, Cohen BD, Coleman K, Christensen JG, Emerson EO, Jakowski A, Hook K, Los G, Moyer JD, Pruimboom-Brees I, Pustilnik L, Rossi AM, Steyn SJ, Su C, Tsaparikos K, Wishka D, Yoon K, Jakubczak JL. 2010. PF-03814735, an orally bioavailable small molecule aurora kinase inhibitor for cancer therapy. Mol Cancer Ther 9:883–894. doi:10.1158/1535-7163.MCT-09-0915. 20354118

[41] Dalva-Aydemir S, Akyerli CB, Yüksel ŞK, Keskin H, Yakıcıer MC. 2019. Toward in vitro epigenetic drug design for thyroid cancer: the promise of PF-03814735, an aurora kinase inhibitor. Omics 23:486–495. doi:10.1089/omi.2019.0050. 31549911

[42] Kimura S. 2010. AT-9283, a small-molecule multi-targeted kinase inhibitor for the potential treatment of cancer. Curr Opin Invest Drugs (London) 11:1442–1449. 21154126

[43] Gripon P, Rumin S, Urban S, Le Seyec J, Glaise D, Cannie I, Guyomard C, Lucas J, Trepo C, Guguen-Guillouzo C. 2002. Infection of a human hepatoma cell line by hepatitis B virus. Proc Natl Acad Sci USA 99:15655–15660. doi:10.1073/pnas.232137699. 12432097 PMC137772

[44] Smith MC, Boutell C, Davido DJ. 2011. HSV-1 ICP0: paving the way for viral replication. Future Virol 6:421–429. doi:10.2217/fvl.11.24. 21765858 PMC3133933

[45] Smith CA, Bates P, Rivera-Gonzalez R, Gu B, DeLuca NA. 1993. ICP4, the major transcriptional regulatory protein of herpes simplex virus type 1, forms a tripartite complex with TATA-binding protein and TFIIB. J Virol 67:4676–4687. doi:10.1128/JVI.67.8.4676-4687.1993. 8392607 PMC237853

[46] Sacks WR, Greene CC, Aschman DP, Schaffer PA. 1985. Herpes simplex virus type 1 ICP27 is an essential regulatory protein. J Virol 55:796–805. doi:10.1128/JVI.55.3.796-805.1985. 2991596 PMC255064

[47] Sedlackova L, Rice SA. 2008. Herpes simplex virus type 1 immediate-early protein ICP27 is required for efficient incorporation of ICP0 and ICP4 into virions. J Virol 82:268–277. doi:10.1128/JVI.01588-07. 17959681 PMC2224399

[48] Kulej K, Avgousti DC, Sidoli S, Herrmann C, Della Fera AN, Kim ET, Garcia BA, Weitzman MD. 2017. Time-resolved global and chromatin proteomics during herpes simplex virus type 1 (HSV-1) infection. Mol Cell Proteomics 16:S92–S107. doi:10.1074/mcp.M116.065987. 28179408 PMC5393384

[49] Garcia G, Jr, Sharma A, Ramaiah A, Sen C, Kohn D, Gomperts B, Svendsen CN, Damoiseaux RD, Arumugaswami V. 2020. Antiviral drug screen of kinase inhibitors identifies cellular signaling pathways critical for SARS-CoV-2 replication. bioRxiv. https://www.biorxiv.org/content/10.1101/2020.06.24.150326v1.

[50] Tolonen N, Doglio L, Schleich S, Krijnse Locker J. 2001. Vaccinia virus DNA replication occurs in endoplasmic reticulum-enclosed cytoplasmic mini-nuclei. Mol Biol Cell 12:2031–2046. doi:10.1091/mbc.12.7.2031. 11452001 PMC55651

[51] Hirota T, Lipp JJ, Toh B-H, Peters J-M. 2005. Histone H3 serine 10 phosphorylation by Aurora B causes HP1 dissociation from heterochromatin. Nature 438:1176–1180. doi:10.1038/nature04254. 16222244

[52] V'kovski P, Kratzel A, Steiner S, Stalder H, Thiel V. 2021. Coronavirus biology and replication: implications for SARS-CoV-2. Nat Rev Microbiol 19:155–170. doi:10.1038/s41579-020-00468-6. 33116300 PMC7592455

[53] Fehr AR, Perlman S. 2015. Coronaviruses: an overview of their replication and pathogenesis. Methods Mol Biol 1282:1–23. doi:10.1007/978-1-4939-2438-7_1. 25720466 PMC4369385

[54] Peng C, Zhou Y, Cao S, Pant A, Campos Guerrero ML, McDonald P, Roy A, Yang Z. 2020. Identification of vaccinia virus inhibitors and cellular functions necessary for efficient viral replication by screening bioactives and FDA-approved drugs. Vaccines 8:401. doi:10.3390/vaccines8030401. 32708182 PMC7564539

[55] Goldenson B, Crispino JD. 2015. The aurora kinases in cell cycle and leukemia. Oncogene 34:537–545. doi:10.1038/onc.2014.14. 24632603 PMC4167158

[56] Schaffer PA, Aron GM, Biswal N, Benyesh-Melnick M. 1973. Temperature-sensitive mutants of herpes simplex virus type 1: isolation, complementation, and partial characterization. Virology 52:57–71. doi:10.1016/0042-6822(73)90398-X. 4372782

[57] Pant A, Dsouza L, Cao S, Peng C, Yang Z. 2021. Viral growth factor- and STAT3 signaling-dependent elevation of the TCA cycle intermediate levels during vaccinia virus infection. PLoS Pathog 17:e1009303. doi:10.1371/journal.ppat.1009303. 33529218 PMC7880457

[58] National Research Council. 2011. Guide for the care and use of laboratory animals, 8th ed. National Academies Press, Washington, DC.

